# To the Members of the Medical Association of Texas

**Published:** 1902-05

**Authors:** 


### TO THE MEMBERS OF THE MEDICAL ASSOCIATION OF TEXAS.

I beg herewith to make the following explanatory statement:

A committee on the revision of the Constitution and By-Laws, appointed by President Taylor Hudson immediately after the adjournment of the Association last April, consists of Drs. J. T. Wilson, Sherman; J. E. Gilcreest, Gainesville; R. F. Miller, Sherman, and H. A. West, Galveston. The committee held its first meeting early in February. Dr. Wilson was elected chairman and Dr. H. A. West secretary. A draft was duly formulated. The second meeting of the committee was called by the secretary to meet at Galveston, April the 19th. Only a portion of the committee were able to attend.

The following report is the result of the deliberations at the last meeting. The only essential difference between the second report and first is upon the basis of representation of component societies in the House of Delegates. It was thought by those present that in view of the great importance of the matter presented, and of the necessity of giving members of the Association an opportunity to consider the same, that the report following should be published in the medical journals of the State, and a copy placed in the hands of every member of the Association. It is earnestly desired that all will carefully study the report so as to be prepared to act upon it intelligently when it is presented before the meeting at Dallas.

The report is unsigned for the reason that copies had to be sent to the journals at once.

The committee will hold a meeting at Dallas on the morning of May the 6th, prior to the opening of the Association.

Respectfully submitted,

H. A. West, Secretary.

### CONSTITUTION.

### ARTICLE I.

*Name of.*

The name of this organization shall be the State Medical Association of Texas.

### ARTICLE II.

*Purposes.*

The purposes of this Association shall be to federate and bring into one compact organization the entire medical profession of the State of Texas, and to unite with similar associations in other States to form the American Medical Association, with a view to the advancement of medical science and art; to the elevation of the standard of medical education, and to the enactment and enforcement of just medical laws; to the promotion of friendly intercourse among physicians; to the guarding and fostering of their material interests, and to the enlightenment and direction of public opinion in regard to the great problems of State medicine, so that the profession shall become more capable and honorable within itself, and more useful to the public in the prevention and cure of disease, and in prolonging and adding comfort to life.

### ARTICLE III.

*Component Societies.*

The State Medical Association of Texas shall be organized into fifteen district associations, embracing contiguous counties as hereinafter defined. The district associations shall be organized into county societies embracing existing societies as they may accept the invitation of the State Association to become its subordinate branches, and such other county societies as the same may be organized. The component societies shall be chartered by the State Association through its House of Delegates.

### ARTICLE IV.

*Composition.*

Section 1. This Association shall consist of delegates and active and associate members.

SEC. 2. *Active Members.*—The active members shall be the members of the component societies.

SEC. 3. *Associate Members.*—The associate members shall consist of members of other scientific bodies who shall make application and shall be elected in due form by the House of Delegates, and who shall be eligible only to read and discuss scientific papers.

SEC. 4. *Delegates.*—Delegates shall be members who are elected in accordance with this Constitution and By-Laws to represent component district societies and shall be regular graduates, active practitioners and conformers to the American Medical Association code of ethics.

SEC. 5. *Guests.*—Distinguished physicians or members of other scientific bodies not residents of this State may become guests of the Association during any annual session upon invitation of the Association or its council, and shall be accorded the privilege of participating in the scientific work.

### ARTICLE V.

### *House of Delegates.*

The House of Delegates shall be the business body of the Association, and shall consist of,

(1) Delegates elected by the component district societies.

(2) (Ex-officio) the President, Vice-Presidents, Secretary, Treasurer, Board of Trustees and Councilors.

### ARTICLE VI.

### *Meetings.*

SECTION 1. *Annual Meeting.*—The Association shall hold an annual meeting at such time and place as may be determined by the House of Delegates, which shall be open to all registered members, delegates and guests; provided, that in case an emergency involving the interests of the Association shall arise in the interim, the President, Vice-Presidents, Secretary and Treasurer, with the Board of Trustees and Councilors, shall have power to change the place and date of meeting, and shall select such place and date as may appear to be most suitable.

### ARTICLE VII.

### *Officers.*

SECTION 1. The officers of the Association shall be a President, three Vice-Presidents, Secretary, Treasurer, Board of five Trustees and Board of Councilors, the latter to be elected by district component associations.

SEC. 2. The President and Vice-Presidents shall be elected annually.

Trustees shall be elected for three years. (See Article XIV.) These officers shall serve until their successors are elected and installed.

SEC. 3. The officers of this Association shall be elected on the morning of the last day of the annual session by a majority secret ballot of the House of Delegates, but no elective delegates shall be eligible to any office named in the preceding section, and no ex-officio delegates shall have a vote in electing them. No person shall be elected to such office who is not in attendance upon that annual session.

### ARTICLE VIII.

*Funds and Expenses.*

SECTION 1. Funds for meeting the expenses of the Association shall be arranged for by the House of Delegates, by an equal per capita assessment upon the component societies of three dollars per member, also voluntary contributions and the profit of publication.

SEC. 2. Funds may be appropriated by the House of Delegates through the Board of Trustees to defray the expenses of the annual session, for publication, to pay traveling expenses of delegates and committee men, publication, and such other purposes as are necessary to promote the interests of the Association and profession.

### ARTICLE IX.

*Referendum.*

The general meeting of the Association may by two-thirds vote order a referendum upon any question pending before the House of Delegates, and that body may by a similar vote submit any question to the General Assembly for final action, a majority vote determining the question.

### ARTICLE X.

*Seal.*

This Association shall have a common seal, with power to change the same.

### ARTICLE XI.

*Amendments.*

The House of Delegates may amend any articles of this Constitution by two-thirds vote of the delegates registered at that annual session, provided that such amendment shall have been presented in open meeting at the previous annual session and that it shall have been sent officially to each component society at least two months before the session at which final action is to be taken.

### ARTICLE XII.

SECTION 1. Members of the component societies shall be privileged to attend all meetings and to take part in the proceedings; provided, that they have no right to vote or participate in discussions before the House of Delegates. They shall receive the Transactions or Journal without charge, and shall receive the protections, benefits and support conferred by the State Association.

SEC. 2. The name of a physician upon the certified roster of a component society which has paid its annual dues shall be prima facia evidence of his right to register at the annual sessions.

SEC. 3. No physician who is under sentence of suspension or expulsion or whose name has been dropped from the roll of members shall be entitled to any of the rights or privileges of this body.

SEC. 4. Resignation must be given in writing to the secretary of the component society and shall not be accepted until all dues are paid.

SEC. 5. No member shall be allowed to resign when under charges which may lead to expulsion.

SEC. 6. When a member in good standing removes to another district he shall transfer his name to the roll of members in the district of his new residence by presenting a duly authenticated card within a year from time of such removal.

### ARTICLE XIII.

*Ethics and Discipline.*

SECTION 1. The Code of Ethics of the American Medical Association shall be the code of this body.

SEC. 2. The adjudication of all questions of ethics and administration of discipline shall be vested in the component and district societies, through their constituted authorities, provided there shall be the right of appeal to the Board of Councilors, whose decision shall be final.

### ARTICLE XIV.

*Board of Trustees.*

There shall be elected by the House of Delegates a Board of Trustees, consisting of five members; at the first election two shall be elected to serve one year, two for two years and one for three years, after which the terms of all shall be for three years, two being elected each year, excepting every third year, when one shall be elected. It is not intended to prohibit the re-election of any member. This Board shall have control, under the House of Delegates, of the finances of the Association, and shall establish a Journal to be called the "Journal of the State Medical Association of Texas," in which the proceedings of the Association and its branches shall be published. This Journal shall be first class in every respect, only legitimate advertisements shall be accepted; it shall be, for the present, monthly; a copy shall be sent to every member, to the President and Secretary of the American Medical Association, an exchange list established; the price to subscribers outside of the Association shall be three dollars per annum.

### ARTICLE XV.

*Organization.*

SECTION 1. The active membership shall be organized into the following censorial districts embracing the counties as hereinafter described and exhibited upon accompanying map:

No. 1. El Paso District, composed of all counties west of the Pecos River, together with Loving, Winkler and Ward counties.

No. 2. Big Springs District, embracing the following counties: Crane, Ector, Midland, Glasscock, Howard, Mitchell, Scurry, Kent, Nolan, Taylor, Jones, Haskell, Knox, King, Dickens, Crosby, Lubbock, Hockley, Cochran, Yoakum, Terry, Lynn, Garza, Gaines, Dawson, Borden, Andrews, Martin, Upton.

No. 3. Pan Handle District, embracing the tier of counties extending east, including Bailey, Lamb, Hale, Floyd, Motley, Cottle, Foard, Hardeman, and all counties north and west of the above tier.

No. 4. San Angelo District, embracing the following counties: Crockett, Sutton, Schleicher, Irion, Tom Green, Sterling, Coke, Runnels, Coleman, Concho, McCulloch, Menard, Kimble and Brown.

No. 5. San Antonio, or Western, District, embracing the following counties: Edwards, Kerr, Bandera, Gillespie, Kendall, Bexar,

Guadalupe, Wilson, Atascosa, Frio, Uvalde, Zavala, Kinney, Medina, Maverick, Dimmit, La Salle and Karnes.

No. 6. Corpus Christi, or Southwest Texas, District, embracing the following counties: Webb, Encinal, Duval, McMillan, Live Oak, Bee, Goliad, Refugio, Aransas, San Patricio, Nueces, Cameron, Hidalgo, Starr and Zapata.

No. 7. Austin District, embracing the following counties: Caldwell, Bastrop, Lee, Williamson, Burnet, Travis, Hays, Blanco, Llano, Mason and San Saba.

No. 8. DeWitt District, embracing the following counties: Gonzales, DeWitt, Victoria, Calhoun, Jackson, Lavaca, Fayette, Colorado, Wharton and Matagorda.

No. 9. South Texas District, embracing the following counties: Brazoria, Fort Bend, Austin, Waller, Montgomery, Harris, Galveston, Chambers and Washington.

No. 10. Southeast Texas District, embracing the following counties: Liberty, San Jacinto, Polk, Tyler, Hardin, Jefferson, Orange, Jasper, Newton, Angelina, Nacogdoches, Shelby, San Augustine and Sabine.

No. 11. Brazos Valley District, embracing the following counties: Burleson, Milam, Robertson, Leon, Madison, Grimes, Walker and Houston.

No. 12. Central Texas District, embracing the following counties: Bell, Lampasas, Mills, Comanche, Hamilton, Coryell, Falls, McLennan, Bosque, Hill, Limestone, Freestone and Navarro.

No. 13. Northwest Texas District, embracing the following counties: Callahan, Shackelford, Throckmorton, Baylor, Wilbarger, Wichita, Archer, Young, Stephens, Eastland, Palo Pinto, Jack and Clay.

No. 14. North Texas District, embracing the following counties: Erath, Hood, Parker, Wise, Montague, Cooke, Denton, Tarrant, Johnson, Ellis, Dallas, Collins, Grayson, Fannin, Hunt, Rockwall, Kaufman, Van Zandt, Rains, Hopkins, Delta and Lamar.

No. 15. Northeast District, embracing the following counties: Cherokee, Rusk, Anderson, Panola, Harrison, Gregg, Smith, Wood, Upshur, Marion, Cass, Morris, Titus, Franklin, Red River and Boyd.

### BY-LAWS.

### ARTICLE I.

SECTION 1. The House of Delegates, in addition to its ex-officio members as defined in Constitution, Article V, shall be composed of delegates elected by the component district associations upon the following basis, viz.: One elective delegate for every one hundred members, and fraction over fifty. Provided, that associations numbering less than 100 members shall be entitled to one in addition to its delegates to its delegate ex-officio.

SEC. 2. This body constituting the legislative and business branch of the Association shall elect all officers and committees not otherwise provided for, and conduct through its committees the business affairs committed to it.

SEC. 3. It shall meet annually at the time and place of the Association meeting; holding its session at such time as will not conflict with the General Assembly. Elective delegates shall hold their office for two years or until their successors are elected and qualified.

SEC. 4. *Officers.*—The President, Vice-Presidents, Secretary and Treasurer of the Association shall be ex-officio the officers of the House of Delegates.

SEC. 5. *The Board of Councilors* shall consist of fifteen members, one to be elected by each component district. They shall serve for three years, but be subject to removal for cause by the House of Delegates. In the event of death or resignation of a member the President of the association in which the vacancy occurs shall have the authority to appoint a successor until the next annual election. To this Board all questions of personal character, including complaints, protests, appeals, ethics, discipline, and credentials, shall be referred immediately after presentation without discussion. The Board shall elect a chairman and secretary, and keep a permanent record of its proceedings and report the same to the House of Delegates before its adjournment.

SEC. 6. Each councilor shall be organizer, peace-maker and chairman of the Board of Censors for his district. He shall visit the various counties as far as practicable at least once a year for the purpose of organizing new county societies, and for improving and stimulating the zeal of members. He shall make annual report of the condition of the profession in each county in his district to the annual session of the House of Delegates. His necessary traveling expenses incurred by such councilor in the line of duties herein imposed may be allowed by the House of Delegates upon a properly itemized statement. This is not intended to be construed as including attendance upon annual sessions of the Association.

### ARTICLE II.

*Quorum of the House of Delegates.*

SECTION 1. Fifteen members will constitute a quorum.

SEC. 2. The order of business at the annual meetings of the House of Delegates shall be as follows:

1. Call to order.
2. Roll call.
3. President's message on Needs of the Association.
4. Secretary's report.
5. Treasurer's report.
6. Report of standing committees.
7. Report of special committees.
8. Unfinished business.
9. New business.
10. Report of Nominating Committee.
11. Election of officers.
12. Reading of minutes and action thereon.

### ARTICLE III.

*Duties of Officers.*

SECTION 1. *President.*—The President shall preside at all general meetings of the Association and of the House of Delegates. He shall appoint committees not otherwise provided for; he shall report the condition and needs of the Association at the annual session of the House of Delegates. He shall deliver an address upon some scientific subject at such time as may be designated by the Committee on Arrangements, and shall fill all vacancies in the officers of the Association which may occur in the interim.

SEC. 2. *Vice-Presidents.*—The Vice-Presidents in regular order, at the request or in the absence of the President, shall temporarily perform his duties. In the event of death, disability, or resignation of the President, the First Vice-President shall act in his place until after the next annual election. If for any reason the First

Vice-President cannot act the second shall do so, and so on in succession. The Vice-Presidents with the President and Secretary shall constitute a committee for the purpose of making special assignments for the discussion of scientific subjects at the annual meetings of the Association, and appointment of Section officers.

SEC. 3. *Secretary.*—The Secretary shall make and preserve accurate minutes of the meeting of the Association, and of the House of Delegates. He shall conduct its correspondence, shall have charge of and preserve all books, papers and property belonging to the Association; keep a correct register of the names of members, dates of admission, and their postoffice addresses. He shall keep a record in his office so far as may be practicable of all legal practitioners within the State, as shown by data furnished him by the secretaries of the several district and county societies; and shall furnish the Secretary of the American Medical Association with a copy of such lists during the year 1903, and each year thereafter with necessary corrections. He shall attend all meetings of committees when required with such documents as may be needed for reference, and report such unfinished business requiring action as may appear upon his books or that may come to his knowledge. As soon after the annual meeting as practicable he shall deliver to the Board of Trustees, or its agent, all such records of proceedings, reports, addresses, papers, and other documents as may have been ordered for publication, either in the general sessions, House of Delegates or Sections. He may nominate a stenographer who shall act as his assistant in the performance of such secretarial and recording duties as may be required during the annual meetings and of the meetings of the district and county societies as far as practicable. The compensation of a stenographer shall be decided by the Board of Trustees. The Secretary shall make an annual report to the Association and House of Delegates. He shall receive for his services such salary as may be fixed by the Board of Trustees. He shall be allowed to draw on the Treasurer for the expenses of his office.

SEC. 4. *Treasurer.*—The Treasurer shall give bond in such amount as may be required by the House of Delegates. He shall collect all funds due the Association from the treasurers of the component societies at least two months before the annual session. He shall, under direction of the Board of Trustees, have the care and management of the fiscal affairs of the Association. He shall keep its accounts and pay all orders drawn on him by the Chairman of the Board of Trustees, signed by the President and countersigned by the Secretary. He shall make an annual report of the finances to the House of Delegates. He shall receive for his services such salary as may be decided upon by the Board of Trustees, subject to approval of the House of Delegates.

SEC. 5. *Duties of Board of Trustees.*—It shall be the duty of this Board, under the House of Delegates, to manage the finances of the Association. Business matters connected with the Journal, superintend the publication of the proceedings, nominate an editor of Journal who shall be subject to removal for cause, prescribe his duties, determine the amount of his salary and his term of office. It shall have power to reject any paper deemed unsuitable for publication. It shall transfer all moneys belonging to the Association in its possession to the Treasurer. (Art. VIII, Section 2, Constitution.) It may pay for actual traveling expenses and hotel expenses of officers and committeemen when it is necessary for them to leave home on Association business ad interim. It shall pay the actual traveling and hotel expenses of the Association delegates to the A. M. A. and meeting of Committee on National Legislation, upon presentation of a properly itemized account of same. It shall audit the accounts of Secretary and Treasurer, and make report to House of Delegates at each annual meeting.

### ARTICLE IV.

### *Standing Committees.*

SECTION 1. There shall be a Committee on Scientific Work, Public Policy and Legislation, Publication, Nomination, Arrangements, and such other committees as may in the opinion of the House of Delegates be necessary.

Such committees shall be elected by the House of Delegates unless otherwise provided.

SEC. 2. The Committee on Scientific Work shall consist of the President, Vice-President and Secretary, as hereinbefore specified.

SEC. 3. The Committee on Public Policy and Legislation shall consist of fifteen members, representing each district society in the State, who shall take into consideration such legislation as may be for the good of the people and medical profession, and urge the enactment of all laws recommended by the House of Delegates.

SEC. 4. The Committee on Publication shall consist of the Board of Trustees as hereinbefore mentioned.

SEC. 5. The Committee on Nomination shall consist of a member from each district society, elected by the House of Delegates, and who shall not be eligible to office, who shall nominate all officers not hereinbefore provided for, in duplicate, and report same to the House of Delegates on the morning of the last day of annual meeting.

SEC. 6. The Committee on Arrangements shall consist of the component societies in the district in which the annual meeting is to be held, which shall by committee of its own selection provide suitable accommodation for the Association, House of Delegates and committees. Its chairman shall report an outline of arrangements and furnish the same to the Secretary or editor for publication.

SEC. 7. The various committees shall hold as many meetings during the year as may be necessary, and a majority of the members shall constitute a quorum. They shall make minutes of their proceedings and report same to the House of Delegates.

### ARTICLE V.

### *Meetings of the Association.*

SECTION 1. *Annual Meeting.*—The Association shall hold an annual meeting at such time and place as may be determined by the House of Delegates, through its Committee on Nominations. The sessions shall be open to all registered members, delegates and guests. In the event of an emergency arising in the interim involving the interests of the Association, the President, Vice-President, Secretary, Treasurer and Board of Trustees shall have authority to make such change in the place and date of meeting as they deem best.

SEC. 2. *Special Meetings.*—Special meetings may be called by the President upon written request of twenty members.

SEC. 3. The journal session of the Association shall not occupy more than two and a half hours of the first morning session, and not exceeding one hour for any other morning session. The remaining morning, afternoon, and first two evening hours shall be devoted to Section work.

SEC. 4. *Quorum.*—Twenty-five members shall constitute a quorum.

SEC. 5. *Order of Business.*—The order of business at the annual meeting shall be as follows: Call to order; roll call; report of chairman of the Committee on Arrangements; special addresses; report from House of Delegates; reports of standing committees; reports of special committees; reading and discussion of papers; President's address; installation of officers; adjournment.

SEC. 6. Roberts's Rules of Order shall govern the parliamentary questions.

### DISTRICT BRANCH ASSOCIATIONS.

### ARTICLE VI.

*Mode of Organization.*

SECTION 1. The district branches shall be composed of the several county societies as hereinbefore specified, and of members living in counties having temporarily no county organization. Counties having ten or more resident physicians are to be organized into societies as soon as possible by the District Councilor. The latter is to make himself familiar with the work of the county societies within his district and to report upon the same at the annual meetings of the House of Delegates.

SEC. 2. District branches shall receive charters from the State Association, and enact constitutions in conformity with that of the State body.

SEC. 3. Component district associations shall be organized upon the same general plan as the State Association, viz.: They are to be divided into (*a*) House of Delegates to be composed of delegates from component county societies upon a basis of one delegate for every fifty members and major fraction thereof; provided, that every county shall be entitled to one delegate. The officers of the district association shall be ex-officio the officers of the House of Delegates.

SEC. 4. Delegates shall be graduates of medicine, legal and active practitioners, and conformers to the Code of Ethics of A. M. A.

SEC. 5. The House of Delegates is to perform the same general functions as that of the similar body of the State Association, viz.: nomination and election of officers; attend to all business affairs and settlement of all questions of ethics and discipline.

SEC. 6. (*b*) The scientific body shall embrace the membership of all component county societies.

SEC. 7. *Officers.*—The officers of the district branches shall be a president, vice-president, secretary, treasurer and councilor. The president and the vice-president shall perform the usual duties as pertain to such office and as may be further defined in the by-laws of each association.

SEC. 8. The secretaries of the district branches shall perform the usual duties pertaining to that office, and shall make an annual report to the House of Delegates, giving a complete list of members in his district, with such other data as may be required by the general Secretary.

SEC. 9. The treasurer shall collect and disburse the funds collected by him, making payment to the general Treasurer as herein provided.

SEC. 10. *Annual Meetings.*—Each district shall so arrange the date of its meetings as to appoint its representatives to the House of Delegates prior to the meeting of the same.

SEC. 11. *Councilors.*—(See Sections 6 and 7, Article I, By-Laws.)

SEC. 12. *Constitution and By-Laws.*—Each district association shall make its own Constitution and By-Laws, the same to be in conformity with those of the State Association, and subject to approval by the House of Delegates.

SEC. 13. All papers worthy of publication, minutes and discussions of the House of Delegates and scientific bodies of component societies becomes the property of the State Association, and shall be delivered as soon as practicable to the general Secretary for publication in the State Journal.

SEC. 14. *Dues.*—The annual dues of members of each component society shall be $3.00; provided, that any society may levy such additional assessments as may be required to meet its own expenses. All members failing to pay dues by April 1st of each year shall stand suspended until reinstated by payment. The treasurer of each component society shall forward the amount collected upon the per capita assessment, with a roster of all officers, members, delegates, and list of non-affiliated physicians in the county, to the general Treasurer at least thirty days in advance of the annual session.

SEC. 15. *Charter.*—The Association shall issue a charter, duly signed by the President and Secretary, to each district association hereinbefore described as soon as the same are ready for organization. Charters are to be placed with the Councilors (to be elected principally by the provisional House of Delegates). The Councilors shall proceed to organize their respective districts as rapidly as possible, as follows: He shall extend an invitation to all county societies already operative to assemble by their representatives at some central place, and proceed to organize the district associations. Those now existing may form the nucleus of the component bodies of this Association.

SEC. 16. *Changes of Counties from One District to Another.*—If the majority of the physicians in a county can show cause for exchange into an adjourning district, the same may be made with the consent of the respective districts, due record of which shall be made and reported to the general Secretary and Treasurer.

### ARTICLE VII.

### *County Societies.*

SECTION 1. All county societies now in affiliation with the State Association, or those that may hereafter be organized in this State, which have adopted principles of organization not in conflict with this Constitution and By-Laws, shall, upon application to the House of Delegates, receive a charter from and become a component part of this Association.

SEC. 2. As rapidly as can be done after the adoption of this Constitution and By-Laws, a medical society shall be organized in every county in the State in which no component society exists, and charters shall be issued thereto.

SEC. 3. Charters shall be issued only upon approval of the House of Delegates, and shall be signed by the President and Secretary of this Association. The House of Delegates shall have authority to revoke the charter of any component county society whose actions are in conflict with the letter or spirit of this Constitution and By-Laws.

SEC. 4. Each county shall judge of the qualifications of its own members, but as such societies are the portals to this Association and to the American Medical Association, every reputable and legally registered physician who is practicing, or who will agree to practice, non-sectarian medicine shall be entitled to membership. Before a charter is issued to any county society, full and ample notice and an opportunity shall be given to every such physician in the county to become a member.

SEC. 5. Only one component medical society shall be chartered in any county. Where more than one county society exists, friendly overtures and concessions shall be made, with the aid of the Councilor for the district if necessary, and all of the members brought into one organization. In case of failure to unite, an appeal may be made to the Council, which shall decide what action shall be taken.

SEC. 6. Any physician who may feel aggrieved by the action of the society of his county in refusing him membership, or in suspending or expelling him, shall have the right of appeal to the Council and to the House of Delegates.

SEC. 7. In hearing appeals the Council may admit oral or written evidence as in its judgment will best and most fairly present the facts, but in case of every appeal, both as a Board and as individual councilors in district and county work, efforts at conciliation and compromise shall precede all such hearings.

SEC. 8. When a member in good standing in a component society moves to another county in the State, his name, upon request, shall be transferred without costs to the roster of the county society into which jurisdiction he moves.

SEC. 9. A physician living on or near a county line may hold his membership in that county most convenient for him to attend, on permission of the society in whose jurisdiction he resides.

SEC. 10. Each county society shall have general direction of the affairs of the profession in the county, and its influence shall be constantly exerted for bettering the scientific, moral and material condition of every physician in the county; and systematic efforts shall be made by each member, and by the society as a whole, to increase the membership until it embraces every qualified physician in the county.

SEC. 11. Physicians residing in counties containing too few to be organized into a society may join the district association by making direct application thereto.

SEC. 12. Scientific work of the Association shall be embraced in the following Sections:

1. General Medicine, Therapeutics, and Dietetics.
2. Gynecology, Obstetrics, and Diseases of Children.
3. Surgery, Genito-Urinary Diseases, and Dermatology.
4. Diseases of the Eye, Ear, Nose, and Throat.
5. Mental and Nervous Diseases, Medical Jurisprudence.
6. State Medicine and Public Hygiene.
7. Pathology and Bacteriology.
8. Chemistry, Materia Medica, and Pharmacy.

SEC. 13. No paper read before the Association shall occupy more than twenty minutes. Discussions shall be limited to five minutes unless unanimous consent be given by the Section for longer time. The author of the paper shall have the right to occupy ten minutes in closing the discussion. No member shall speak twice on the same subject without permission.

